# Trans-catheter closure of ASD and abnormal connection of left pulmonary vein to vertical vein: A case report

**DOI:** 10.1097/MD.0000000000031011

**Published:** 2022-10-21

**Authors:** Mahmoud Mohamadzadeh Shabestari, Abdollah Kerachian, Hoorak Poorzand, Ali Eshraghi, Faeze Keihanian

**Affiliations:** a Cardiovascular Department, Imam Reza Hospital, Faculty of Medicine, Mashhad University of Medical Sciences, Mashhad, Iran; b Cardiovascular Department, Imam Reza Hospital, Faculty of Medicine, Mashhad University of Medical Sciences, Mashhad, Iran; c Vascular and Endovascular Surgery Research Center, Imam Reza Hospital, Faculty of Medicine, Mashhad University of Medical Sciences, Mashhad, Iran; d Cardiovascular Department, Imam Reza Hospital, Faculty of Medicine, Mashhad University of Medical Sciences, Mashhad, Iran; e Cardiovascular Department, Imam Reza Hospital, Faculty of Medicine, Mashhad University of Medical Sciences, Mashhad, Iran; f Pharmaceutical Research Center, Mashhad University of Medical Sciences, Mashhad, Iran.

**Keywords:** device closure, partial anomalous pulmonary venous connection, vertical vein

## Abstract

Partial anomalous pulmonary venous connection is a rare congenital anomaly in which one or more pulmonary veins are connected to the venous circulation leading to left to right heart shunt. Although correction of anomalous pulmonary venous connection is achieved through surgery, there are rare instances where the abnormal pulmonary vein has dual connection to both left atrium and the major systemic veins. Under these circumstances, catheter-based treatment might become a feasible option.

**Patient concerns:**

A 22-year-old female presented with exertional dyspnea, holo-systolic murmur in left sternal border, and fixed splitting of S2 in examination.

**Diagnosis:**

The patient was diagnosed with secundum type atrial septal defect (ASD) and dual drainage of left upper pulmonary vein.

**Interventions:**

The patient was candidate for device closure. Under TEE guidance, occluder devices were deployed in the upper part of vertical vein and subsequently in place of ASD.

**Outcomes:**

Echocardiogram in the next day showed complete occlusion of flow through the vertical vein and ASD. Dual antiplatelet was prescribed on discharge. Follow-up echocardiography after 3 months showed obvious improvement in RV size. Due to suspicion for clot formation, TEE was done and thrombosis with approximate length of extension of 15 mm was detected back to the device. The patient is following for 5 years. Repeated TEE after 2 years did not show any change in the burden of clot.

**Lessons:**

For comprehensive evaluation of patients with ASD, assessment of pulmonic veins is crucial and in the presence of a vertical vein, the dual drainage of pulmonic veins should be considered.

## 1. Introduction

Partial anomalous pulmonary venous connection (PAPVC) is a rare congenital anomaly in which pulmonary veins carry blood from the lungs to the right side of the heart. The condition has a prevalence of 0.4% to 0.7%, it is frequently diagnosed as an incidental finding.^[1,2]^ Herein we described an adult patient with atrial septal defect (ASD), in which the left upper pulmonary veins (LUPV) drained into the innominate vein which were successfully obstructed by occlusion device.

## 2. Case presentation

**Figure 1.**
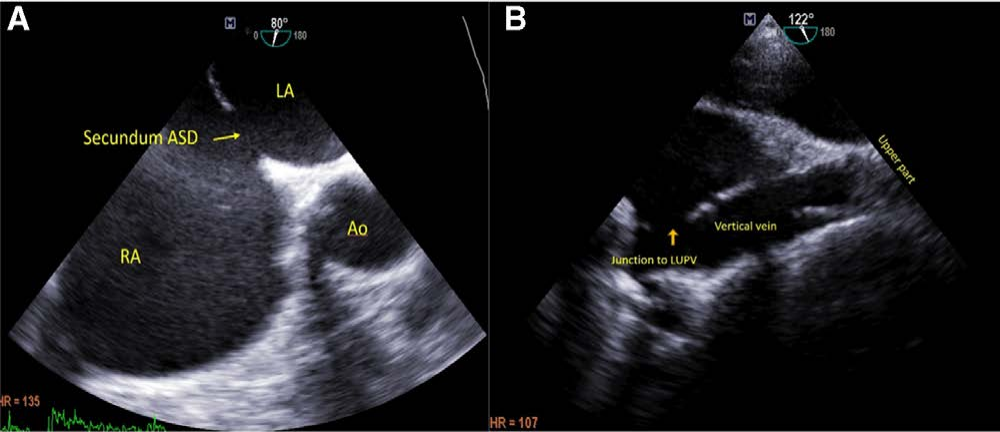
Transesophageal echocardiography images. (a) Two-dimensional TEE image shows atrial septal defect and (b) vertical vein before device closure. Ao = aorta, LA = left atrium, LUPV = left upper pulmonary vein, RA = right atrium, TEE = trans esophageal echocardiography.

**Figure 2.**
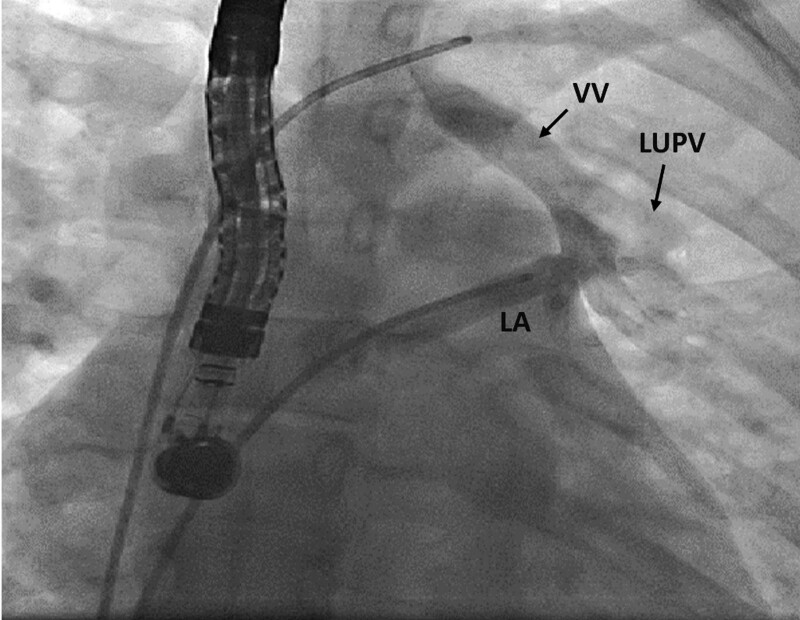
The catheter was positioned in the left pulmonary vein, where the angiography was performed. LA = left atrium, LUPV = left upper pulmonary vein, VV = vertical vein.

A 22-year-old female was referred to our clinic for exertional dyspnea since one month ago. Physical examination defined holo-systolic systolic murmur in left lower sternal border with fixed splitting of S2. Twelve-lead electrocardiogram showed normal sinus rhythm with incomplete right bundle branch block. Transthoracic echocardiography (TTE) was done. Right ventricle was dilated with preserved systolic function. Paradoxical septal motion was noted. There was mild to moderate tricuspid regurgitation. Secundum ASD was seen with left to right shunt. The estimated systolic pulmonary artery pressure was 45 mmHg. A vertical vein was detected traversing at the left side of the thoracic descending aorta and draining into innominate vein. The calculated Qp/Qs was 1.85. In transesophageal echocardiography (TEE), ASD size was 16 mm, with suitable rims (except to the antero-superior rim). Left and right pulmonic veins were seen draining into left atrium. LUPV seemed to be narrow with turbulent flow passing the vein (Peak velocity: 1.2 m/s, mean pressure gradient: 5 mmHg). The vertical vein and the abnormal drainage of LUPV could be clearly seen in TEE (Fig. 1). Diameter of LUPV and the site of connection to the vertical vein was about 5-mm in 2D TEE. No other associated abnormality was reported. The patient underwent cardiac catheterization that confirmed secundum type ASD and the connection of the vertical vein to both innominate vein and the left atrium via the left upper pulmonary vein (Fig. 2).

**Figure 3.**
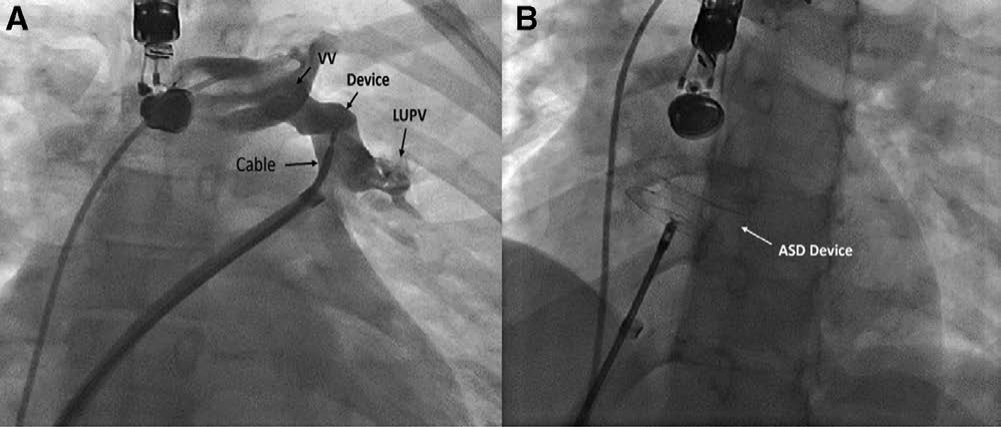
Angiogram demonstrate (a) VSD occluder device in proper position in the abnormal connection without significant residual flow into the left atrium and (b) ASD device. ASD = atrial septal defect, LUPV = left upper pulmonary vein, VSD = ventricular septal defect, VV = vertical vein.

**Figure 4.**
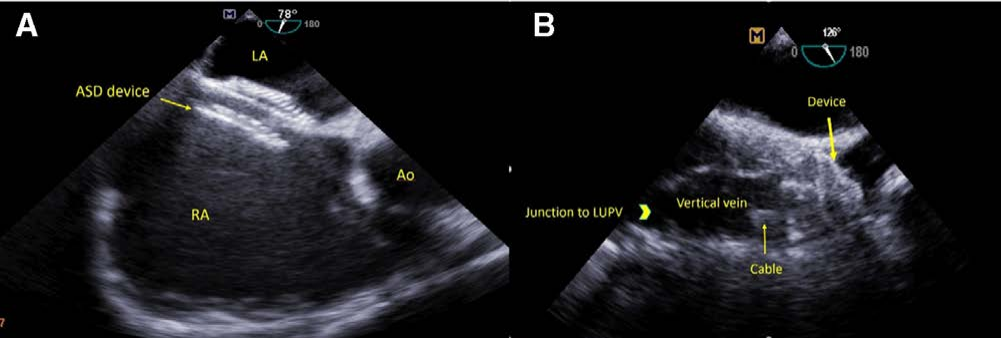
Transesophageal echocardiography images. The images show (a) the ASD device deployed in an appropriate position and (b) the VSD occluder device with complete occlusion of the vertical vein. Ao: aorta, ASD = atrial septal defect, LA = left atrium, LUPV = left upper pulmonary vein, RA = right atrium, VSD = ventricular septal defect.

The patient was referred for device closure. On admission blood pressure was 110/60 mmHg, heart rate was 85 beats/min, and respiratory rate was 18 breaths/min and O2 saturation at room temperature was 94%. After local anesthesia and placing two 6F sheaths in femoral artery and femoral vein, full oximetry run was done. Left to right shunts in level of pulmonary vein to abnormal connection and in ASD level was proved. The patient deeply sedated. Then 6f sheath of vein access was replaced with 10F sheath. Under TEE guidance, muscular ventricular septal defect occluder (12 mm) was deployed in the upper part of vertical vein, at its connection to innominate vein (Fig. 3a). Contrast injection proved optimal occlusion. Then, under TEE guidance and using the same 10F sheath, ASD occluder (Figulla® Flex II ASD, 18 mm) was deployed in place of ASD (Fig. 3b). TEE proved eliminated flow of the vertical vein and proper position of devices with no compressive effect on adjacent structures and no clot (Fig. 4).

Follow-up echocardiogram the next day showed complete occlusion of flow through the vertical vein and ASD. Dual antiplatelet with ASA and Clopidogrel were prescribed on discharge.

**Figure 5.**
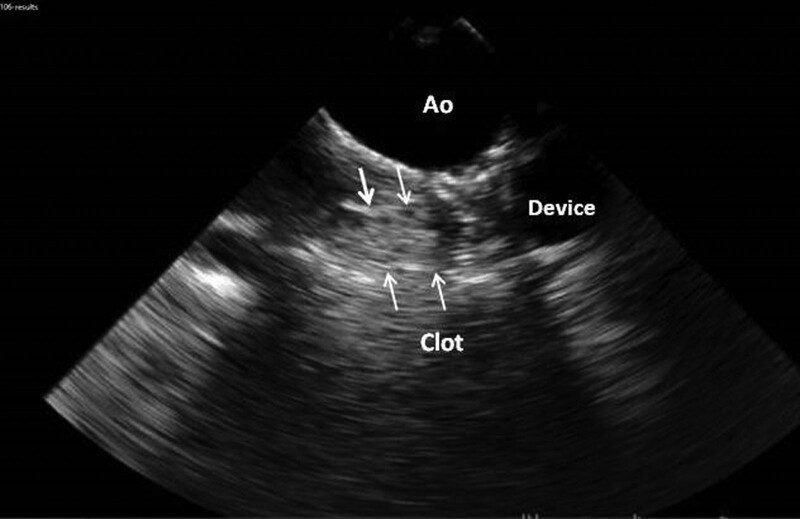
Transesophageal echocardiography images. It shows the clot formation back to the device in the vertical vein.

Trans-thoracic echocardiography was done three months later. There was obvious improvement in right ventricle size. Haziness was suspected behind the device in the vertical vein, which was confirmed in TEE, and ascribed to the clot formation back to the device with approximate length of 15 mm (Fig. 5). Turbulent flow in the narrow LUPV was seen with peak velocity of flow reaching to 1.7 m/s. Pulmonary artery pressure was normal (#20 mmHg). The patient is following for 5 years. She is symptom free. Repeated TEE after 2 years did not show any change in the burden of clot. Single antiplatelet therapy with ASA continued. On annual TTE, the turbulent flow in LUPV was still evident. Pulmonary artery pressure remained in normal limits.

## 3. Discussion

PAPVC is a relatively uncommon congenital anomaly characterized by one or more pulmonary veins draining into a systemic vein or the right atrium rather than the left atrium. Overall, PAPVC is symptomatic and often co-exists with ASD, in 80% to 90% of the cases.^[3]^ Dual drainage of the pulmonary veins to both a systemic vein and LA is rare and difficult to estimating the incidence, because most of these patients are asymptomatic.^[4]^ Trans-catheter closure of the abnormal venous connection is certainly the treatment of choice for PAPVC with dual drainage, although successful surgical ligation of the vertical vein has been reported.^[5]^ The closure could be attained by coil or Amplatzer duct occluder or vascular plugs.^[6]^ The type of closure device used is determined by the anatomical size and shape of the abnormal venous connection.^[4]^ We used a muscular ventricular septal defect occluder in our patient providing complete occlusion of vertical vein; concurrently utilized Amplatzer® occluder for secundum ASD. Performing a balloon occlusion test in the vertical vein, before implanting the device has been suggested to confirm the adequacy of the alternative drainage. Device closure could not be done in cases with ≥10 mmHg increase in pulmonary venous pressure.

In this case, the flow velocity of pulmonic vein was increased from 1.2 to 1.7 m/s after the device closure; but pulmonary artery pressure did not increase during 5-year follow up. Device thrombosis would be a possible serious complication. Optimal antiplatelet or antithrombotic medication have been proposed to enhance safe and complete endothelial coverage of the implanted device. We encountered clot formation behind the implanted device while the patient was on dual antiplatelet therapy, which was ascribed to stagnation of flow. Conservative management with periodic follow up was selected and there was not further extension or increase in the burden of clot on periodic TTE studies. The patient is still receiving ASA a single antiplatelet therapy.^[7]^

Although TTE is usually unable to detect thrombus formation on the device, routine TEE examination is not suggested in adult patients in many centers, and TTE as an imaging tool might be sufficient. TEE could be performed in cases when transthoracic echocardiography suggests thrombosis or when transthoracic images are suboptimal.^[7]^

## 4. Conclusion

For comprehensive evaluation of patients with ASD, assessment of pulmonic veins is crucial and in the presence of a vertical vein, the dual drainage of pulmonic veins should be considered. Trans-catheter interventions could be a safe and efficient treatment of such cases and the thrombus formation after device closure of the vertical vein could be managed conservatively.

## Acknowledgements

We all thank nurses of Imam Reza Hospital helped us in different stages of patient evaluations.

## Author contributions

M.M.S. and H.P. analyzed and interpreted the patient data regarding the cardiovascular disease and managed the patient. H.P. wrote the first draft of the manuscript. A.K. helped in data gathering and diagnosis patient in echocardiographic evaluation. A.E. helped in patient catheterization and management. F.K. analyzed and interpreted the patient data and wrote the first draft of the manuscript. All authors read and approved the final manuscript.

**Conceptualization:** Mahmoud Mohamadzadeh Shabestari, Abdollah Kerachian, Hoorak Poorzand.

**Data curation:** Abdollah Kerachian, Faeze Keihanian.

**Investigation:** Abdollah Kerachian, Hoorak Poorzand, Ali Eshraghi, Mahmoud Mohamadzadeh Shabestari.

**Methodology:** Hoorak Poorzand, Ali Eshraghi.

**Project administration:** Mahmoud Mohamadzadeh Shabestari.

**Writing – original draft:** Hoorak Poorzand, Faeze Keihanian.

**Writing – review & editing:** Hoorak Poorzand, Faeze Keihanian.

## References

[1] Javangula K Cole J Cross M. An unusual manifestation of left partial anomalous pulmonary venous connection. Interact Cardiovasc Thorac Surg. 2010;11:846–7. 20805252 10.1510/icvts.2009.231100

[2] Senocak F Ozme S Bilgiç A. Partial anomalous pulmonary venous return. Evaluation of 51 cases. Jpn Heart J. 1994;35:43–50. 8201780 10.1536/ihj.35.43

[3] Tourmousoglou C Kalogeropoulou C Koletsis E. Right upper lobe partial anomalous pulmonary venous connection. Case Rep Vasc Med. 2014;2014:249896. 24716092 10.1155/2014/249896 PMC3971852

[4] Ari S Ari H Vatansever F. Transcatheter treatment of partial anomalous pulmonary venous connection to left subclavian vein. Turk Kardiyoloji Dernegi arsivi. 2020;48:771–4. 33257612 10.5543/tkda.2020.44376

[5] Saedi S Saedi T. Catheter intervention for abnormal pulmonary venous drainage. Egypt Heart J. 2018;70:125–7. 30166894 10.1016/j.ehj.2018.01.003 PMC6112374

[6] Wilson W Horlick E Benson L. Successful transcatheter occlusion of an anomalous pulmonary vein with dual drainage to the left atrium. Catheter Cardiovasc Interv. 2015;85:1212–6. 25384927 10.1002/ccd.25734

[7] Olasinska-Wisniewska A Grygier M. Antithrombotic/Antiplatelet treatment in transcatheter structural cardiac interventions-PFO/ASD/LAA occluder and interatrial shunt devices. Front Cardiovasc Med. 2019;6:75. 31231662 10.3389/fcvm.2019.00075 PMC6568033

